# Novel Non‐Hyperemic Coronary Physiology Indices for Vessel Longitudinal Analysis

**DOI:** 10.1002/ccd.70065

**Published:** 2025-08-05

**Authors:** Simone Fezzi, Guy F. A. Prado, Luigi Alberto Iossa, Daixin Ding, Elisabetta Pianezzola, Federico Cesar Vigo, Paolo Alberto Del Sole, Verdiana Galli, Stefano Andreaggi, Domenico Tavella, Shengxian Tu, Gabriele Pesarini, Flavio Ribichini, Roberto Scarsini

**Affiliations:** ^1^ Division of Cardiology, Department of Medicine University of Verona Verona Italy; ^2^ Department of Clinical and Molecular Medicine Sapienza University Rome Italy; ^3^ Department of Cardiology Ren Ji Hospital, School of Medicine and School of Biomedical Engineering Shanghai Jiao Tong University Shanghai China

**Keywords:** coronary artery disease, Instantaneous Free‐ratio Pullback Pressure Gradient, percutaneous coronary intervention, quantitative flow ratio

## Abstract

**Background:**

Physiological pattern of coronary artery disease, whether focal or diffuse, is critical in guiding physicians during the decision‐making process for percutaneous coronary interventions.

**Aims:**

This study introduces two novel non‐hyperemic coronary physiology indices designed for longitudinal vessel analysis.

**Methods:**

In this prospective observational study, 415 patients underwent pressure‐wire functional assessments using instantaneous wave‐free ratio (iFR) pullback traces between March 2015 and November 2023 at Verona University Hospital. After applying exclusion criteria, the final study population comprised 198 patients with 209 intermediate coronary lesions. Vessels were qualitatively categorized as focal, diffuse, mixed‐focal, or mixed‐diffuse based on expert panel interpretation. The novel iFR Pullback Pressure Gradient (iPG) was derived from pullback curves to quantify the atherosclerotic pattern along the vessel. The local severity of lesions was assessed using the instantaneous iFR gradients per unit of length (diFR/ds). Additionally, based on Murray law‐based Quantitative Flow Ratio (µFR), both µFR‐PPGi and dµFR/ds were computed, to evaluate their correlation with iFR‐derived metrics.

**Results:**

The optimal iPG threshold for defining focal disease was 0.71 (Youden index = 0.456), demonstrating good accuracy in predicting the predominant disease pattern (AUC 0.785, *p* < 0.001). iPG provided an accuracy of 72%, with a sensitivity, specificity, positive predictive value, negative predictive value of 86.5%, 59%, 67.6%, 81.5%, respectively. The µFR‐PPGi and dµFR/ds showed significant correlations with iPG and diFR/ds, respectively (r = 0.238, *p* < 0.001; r = 0.528, *p* < 0.001).

**Conclusions:**

iPG and diFR/ds are novel quantitative indices for assessing physiological patterns of coronary artery disease, without the need for hyperemia induction. Moderate agreement between angio‐ and iFR‐based indices was found.

**Abbreviations**
ACS: acute coronary syndrome
AUC: area under the curve
BMI: body mass index
CABG: coronary artery bypass graft
CAD: coronary artery disease
diFR/ds: iFR gradient per unit length
DS%: diameter stenosis percentage
dµFR/ds: instantaneous µFR gradient per unit length
FFR: fractional flow reserve
iFR: instantaneous wave‐free ratio
iPG: iFR Pullback Pressure Gradient
NPV: negative predictive value
PAD: peripheral artery disease
PCI: percutaneous coronary intervention
PPGi: pullback pressure gradient index
PPV: positive predictive value
PW: pressure wire
ROC: receiver operating characteristic
RVD: reference vessel diameter
STEMI: ST‐elevation myocardial infarction
µFR: Murray's law–derived quantitative flow ratio
µFR PPGi: µFR Pullback Pressure Gradient

## 1 Introduction

Relieving symptoms and the burden of myocardial ischemia is the primary goal of revascularization in patients with chronic or acute obstructive coronary artery disease (CAD) [1, 2]. The physiological pattern of CAD significantly influences percutaneous coronary intervention (PCI) outcomes, with diffuse disease being associated with suboptimal post‐PCI physiology, increased risk of periprocedural myocardial infarction, and higher burden of residual symptoms at follow‐up [3, 4]. The classification of disease as focal or diffuse has been formalized using physiological indices such as the hyperemic PPG, although differentiating between these patterns in practice may remain challenging due to intermediate or mixed presentations [5]. To address these limitations, several mathematical metrics have been developed to quantitatively classify physiological patterns, including fractional flow reserve (FFR) gradient per unit time (dFFR[t]/dt) and pullback pressure gradient index (PPGi). These metrics were applied to determine the disease severity along the course of the vessel and predict post‐PCI outcomes. Both are calculated based on pressure‐wire (PW)‐pullback performed during continuous hyperemia. PPGi quantitatively measures the physiological distribution of coronary plaques along the vessel and is capable of distinguishing between focal and diffuse disease. On a scale from 0 to 1, low PPGi is suggestive of diffuse disease, high PPGi of focal. dFFR[t]/dt reflects the local physiological disease severity, distinguishing between minor or major pressure drops on the physiological map of the vessel [5, 6, 7, 8]. PPGi and dFFR/dt provide a second dimension to epicardial coronary physiology, and their clinical importance is increasingly recognized. While FFR guides revascularization decisions, PPGi and dFFR/dt provide insights on the potential physiological benefits achievable with PCI [9]. However, the use of FFR remains limited in clinical practice, due to procedural complexities, increased costs and the need for hyperemia induction. Consequently, many operators favor alternative methods, such as non‐hyperemic pressure ratios or angiography‐derived surrogates [10].

Recently, angiography‐derived physiological indices able to compute FFR from coronary angiography have been endorsed by international guidelines [11]. Quantitative measures of coronary disease spatial distribution derived from quantitative flow ratio (QFR) virtual pullback traces have been already validated [12, 13].

In this study we aimed to develop two novel indices of vessel longitudinal analysis based on instantaneous wave free ratio (iFR) scout‐pullback: the Instantaneous Free‐ratio Pullback Pressure Gradient (iPG) and the instantaneous iFR gradients per unit of length (diFR/ds). Secondly, we sought to test the accuracy of iPG in characterizing the disease pattern, compared to a consensus of expert operators based on qualitative pullback traces interpretation. Lastly, we compared the iPG and diFR/ds classification with Murray's law quantitative flow ratio (µFR)‐derived indices.

## 2 Methods

### 2.1 Study Population

Consecutive patients with intermediate coronary lesions who underwent vessel longitudinal analysis with the iFR Scout pullback system (Philips Medical Systems, Best, the Netherlands) at Verona University Hospital (Verona, Italy) between March 2015 and November 2023 were included in the study. The analysis of coronary physiology traces was centralized and performed blinded by expert independent operators.

Patients with unavailable or poor‐quality coronary angiograms, suboptimal quality of pressure traces, severe tortuosity, as well as those with previous coronary artery bypass grafting, angiographically identifiable myocardial bridging, or collaterals, were excluded. Vessels with a distal iFR > 0.95 were considered physiologically unobstructed and were also excluded. The final study population comprised 198 patients with 209 vessels.

### 2.2 Wire‐Based Physiological Assessments (FFR and iFR)

Coronary physiology assessment was performed using the Verrata Plus or Omniwire pressure wire (Philips Volcano, San Diego, CA), following standardized guidelines and the local institutional protocol [14].

Briefly, after normalization of the PW at the ostium of the interrogated vessel and consequent positioning in the distal segment, fluoroscopy was used and recorded to confirm wire's position, and the iFR spot value was measured. Subsequently, FFR was assessed under steady state hyperemia, induced by systemic administration of adenosine (140 mcg/kg/min) or by intracoronary bolus injection of adenosine (100 μg for the right coronary artery and 200 μg for the left coronary artery). After the complete remission of the hyperemic state, the iFR pullback evaluation was performed manually using the iFR Scout system (Philips Medical Systems, Best, The Netherlands). Operators were instructed to maintain a controlled pullback pace of approximately 0.5–1 mm/s and perform the pullback manoeuvre over 20–30 s. ﻿The presence of a significant pressure‐wire drift (+/− 0.03) was excluded as the pressure sensor reached the ostium of left main or right coronary artery. Cases were included exclusively when pullback started at an adequately distal point of the vessel or if fluoroscopy was provided to confirm the distal wire position. To ensure measurement reliability, multiple recordings were obtained for each assessment. Clinically recommended cut‐off values (FFR ≤ 0.80 and iFR ≤ 0.89) were applied.

### 2.3 Angiography‐Derived Coronary Physiology Assessment

Angiography‐derived coronary physiology assessment was performed using the AngioPlus Core Software (Pulse Medical, Shanghai, China) to derive the Murray's law quantitative flow ratio (µFR).

**FIGURE 1:**
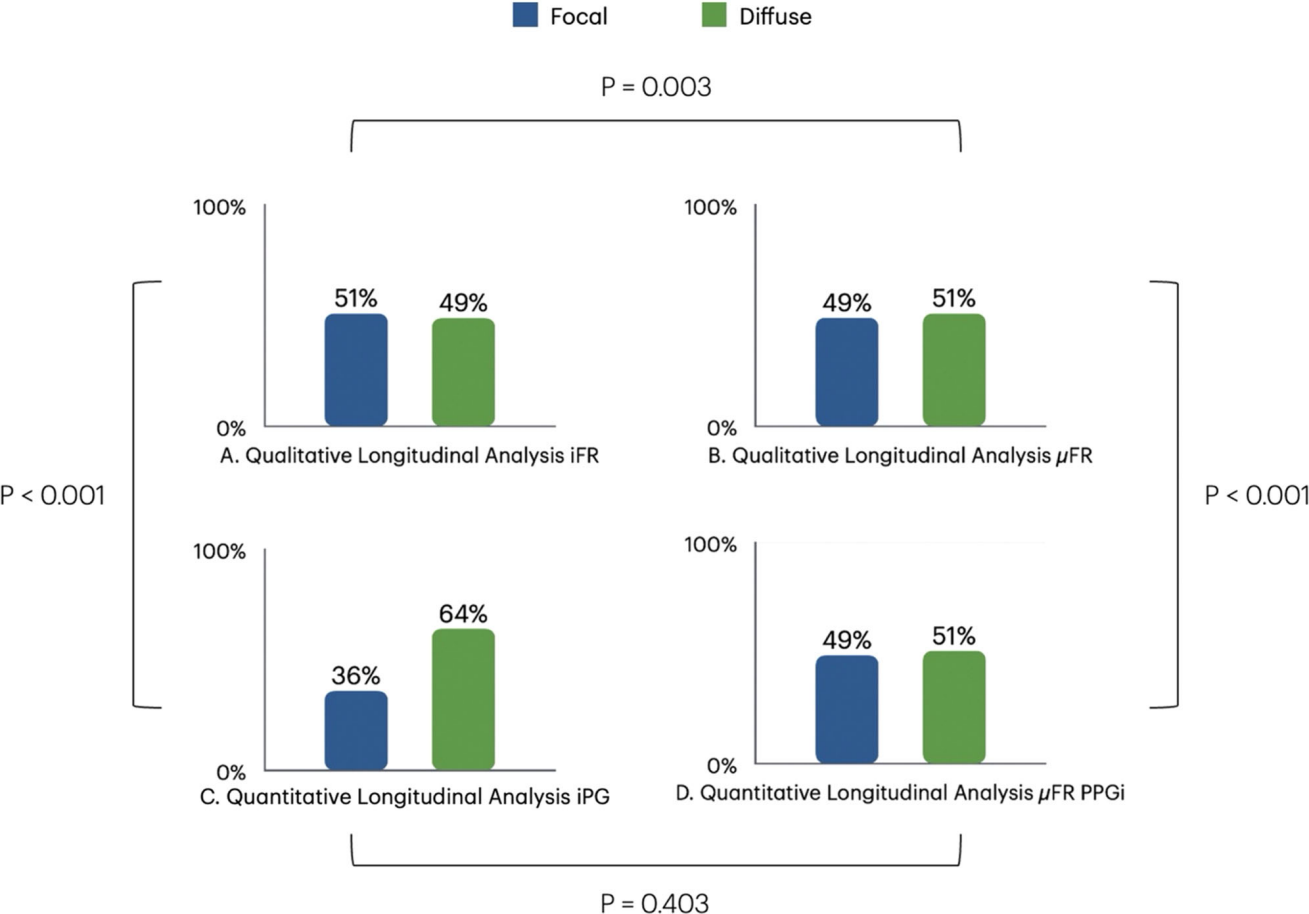
Physiological pattern of disease classification across different pullback analysis methods. Proportion of vessels classified as focal (focal plus mixed‐focal is reported in blue) or diffuse (diffuse plus mixed‐diffuse is reported in green), at the qualitative longitudinal interpretation from experts based on iFR pullback curves (A) and µFR virtual pullback curves (B), and at the quantitative assessment with iPG (C) and µFR PPGi (D). Panels A and B show similar distributions of focal and diffuse patterns in qualitative analyses of iFR (51% vs. 49%) and μFR (49% vs. 51%). Panel C (quantitative iPG) highlights a higher prevalence of diffuse disease (64% vs. 36%), while Panel D (quantitative μFR PPGi) mirrors the qualitative distributions (49% vs. 51%). Significant correlations are observed between qualitative and quantitative methods (*p* < 0.001) and between qualitative iFR and μFR (*p* = 0.003), but not between quantitative evaluation based on iPG and μFR PPGi (*p* = 0.403). These findings highlight the complementary roles of qualitative and quantitative approaches, with quantitative methods like iPG providing greater sensitivity in identifying diffuse disease patterns. iFR, instantaneous wave‐free ratio; iPG, Instantaneous free‐ratio pullback pressure gradient index; µFR, Murray's law quantitative flow ratio; µFR PPGi, µFR pullback pressure gradient index. [Color figure can be viewed at wileyonlinelibrary.com]

µFR assessment was performed off‐line by expert certified operators blinded to invasive coronary physiology measurements and to clinical data. µFR computations adhered to the workflow provided by Pulse Medical, with the single view 2D µFR applied in all cases [15, 16]. To ensure consistency and accuracy in vessel length measurements across cases, we retrospectively matched the distal pressure‐wire position at the start of the iFR pullback, recorded by fluoroscopy, with the corresponding anatomical segment identified by µFR‐based reconstructions. These reconstructions provide both physiological data and QCA‐derived anatomical vessel length. The µFR‐derived length was then used as a standardized spatial reference for all subsequent analyses, including iFR and µFR pullbacks (Supporting Information S1: Figure 1). The virtual pullback was automatically generated by the AngioPlus Core Software. Conventional validated cut‐off values for ischemia (μFR ≤ 0.80) were applied [17].

### 2.4 Pullback Data Extractions

**FIGURE 2:**
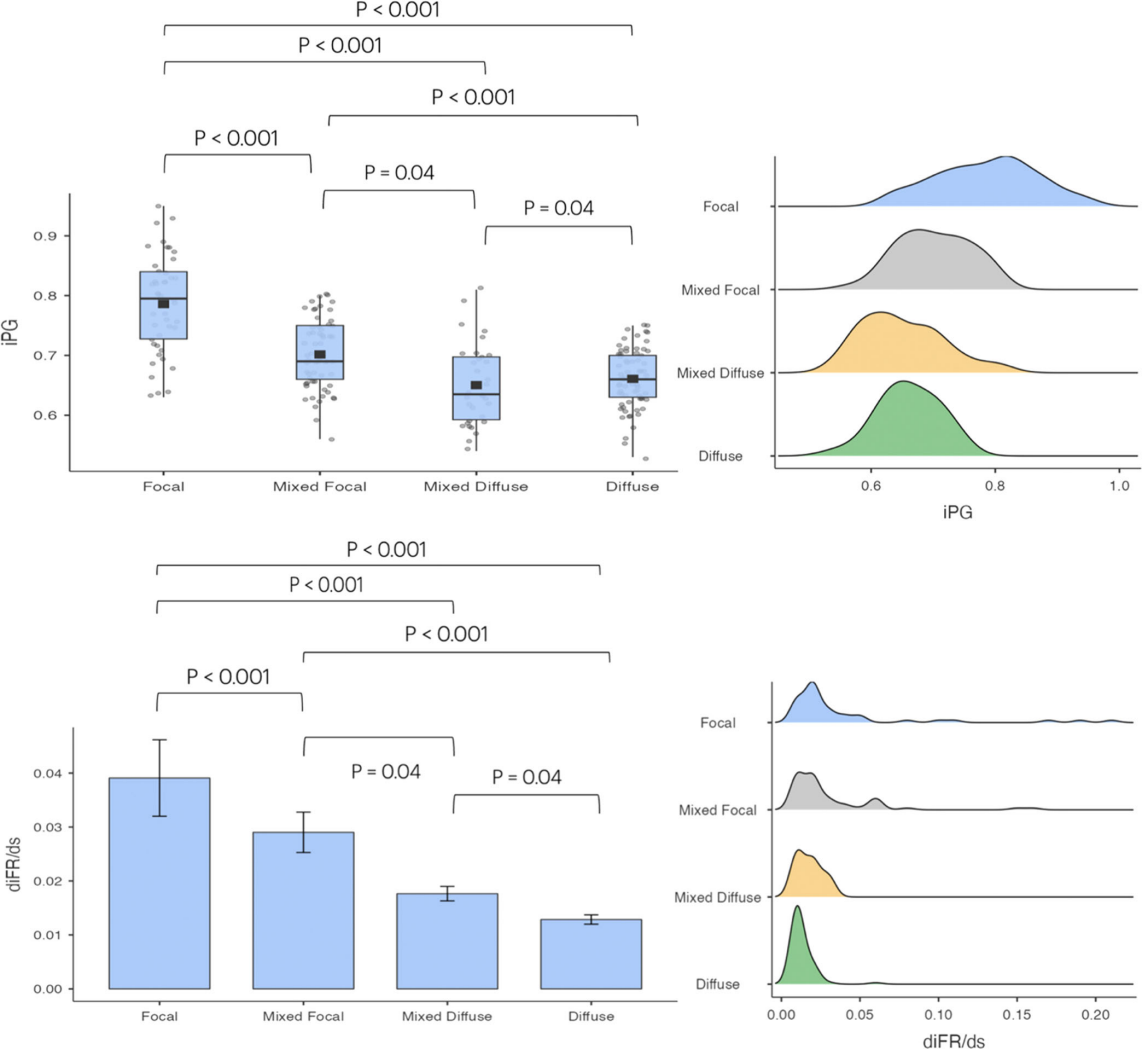
Distribution of iPG and diFR/ds indices across different disease patterns. Distribution of iPG and diFR/ds indices across different disease patterns (Focal, Mixed Focal, Mixed Diffuse, and Diffuse) as determined by qualitative longitudinal iFR analysis. In the upper panel, iPG data are represented through boxplot (on the left) and density plot (on the right). Significant differences are observed among all groups, with focal lesions displaying the highest iPG values and diffuse disease showing the lowest (*p* < 0.001 for most comparisons, *p* = 0.04 between mixed focal and mixed diffuse, and mixed diffuse and diffuse). In the lower panel, diFR/ds values distribution is represented in a bar chart (on the left) and in a density plot (on the right). Focal and mixed focal lesions show significantly higher diFR/ds values compared to mixed diffuse and diffuse disease (*p* < 0.001 for most comparisons, *p* = 0.04 between mixed categories). The density plot for diFR/ds in predominantly focal lesions exhibits a secondary hump, which likely reflects the heterogeneity within this category. This may correspond to subgroups of focal lesions with varying hemodynamic impacts: those with mild pressure gradients (lower diFR/ds values) and those with steeper gradients localized in short segments (higher diFR/ds values). Alternatively, anatomical or technical factors, as well as overlaps with mixed patterns, might contribute to this distribution. These visualizations underline the complementary roles of iPG and diFR/ds in differentiating focal, mixed, and diffuse disease patterns, with diFR/ds providing a more localized assessment of pressure gradients along the vessel. diFR/ds, Instantaneous iFR gradients per unit of length; iFR, instantaneous wave‐free ratio; iPG, Instantaneous free‐ratio pullback pressure gradient index. [Color figure can be viewed at wileyonlinelibrary.com]

Graphical data from both invasive iFR pullback and virtual µFR pullbacks were extracted using WebPlotDigitizer (Automeris LLC), an open‐source software designed to digitize plots from image files. High‐resolution screenshots of the pullback curves were uploaded and manually calibrated using a Cartesian coordinate system. The Y‐axis corresponded to iFR or µFR values, while the X‐axis represented the total vessel length as derived from µFR‐based anatomical reconstructions. Data points were extracted at fixed 1 mm intervals using the X step/interpolation algorithm (X‐step function, ΔX = 1.0 mm). This approach was applied consistently across all pullbacks, providing high‐resolution, reproducible datasets for subsequent quantitative analysis (Supporting Information S1: Figure 2). This method has been previously validated and used in cardiovascular research, with good reproducibility and accuracy [18].

### 2.5 Instantaneous Free‐Ratio Pullback Pressure Gradient (iPG) and the Instantaneous iFR Gradient Per Unit Length (diFR/ds)

The Instantaneous Free‐ratio Pullback Pressure Gradient index (iPG) was developed based on FFR‐derived PPGi formula previously described [5]. iPG provides a continuous metric that integrates the magnitude of the maximum pressure drop over a 20 mm segment and the extent of functional disease across the interrogated vessel. The iPG is calculated as follows:

$$\frac{\left[\frac{{\mathrm{Max iFR}\;P\text{PG}}_{20\mathrm{mm}}}{{∆\text{iF}R}_{\mathrm{vessel}}}+\left(1-\frac{{\mathrm{Length with functional disease}}_{\left(\mathrm{mm}\right)}}{{\mathrm{Total vessel length}}_{\left(\mathrm{mm}\right)}}\right)\right]}{2}$$

MaxPPG_20mm_ corresponds to the maximum gradient observed over a continuous 20 mm segment of the vessel. ΔiFR_vessel_ represents total pressure drop along the vessel. Length of functional disease refers to the cumulative number of 1 mm segments along the vessel exhibiting a continuous pressure drop equal to or greater than 0.0015 per mm. Total vessel length used in the formula was defined as the anatomical length derived from µFR reconstructions.

High iPG values (close to 1) suggest predominantly focal disease, whereas low values (close to 0) predominantly diffuse disease.

The local physiological disease severity was estimated using the instantaneous iFR gradient per unit of length (diFR/ds), defined as the maximum iFR gradient in 1 mm. The mean value of diFR/ds was applied to identify the presence or the absence major gradients.

### 2.6 µFR‐Derived Pullback Pressure Gradient Index (µFR‐PPGi) and Instantaneous µFR Gradient Per Unit of Length (dµFR/ds)

Quantitative measures of longitudinal analysis were derived from the µFR virtual pullback as previously described [20]. Briefly, µFR‐PPGi was derived applying the PPGi formula to the µFR virtual pullback as follows:

$$\frac{\left[\frac{{\mathrm{Max}\mathrm{µFR PPG}}_{20\mathrm{mm}}}{{∆\mathrm{µF}R}_{\mathrm{vessel}}}+\left(1-\frac{{\mathrm{Length with functional disease}}_{\left(\mathrm{mm}\right)}}{{\mathrm{Total vessel length}}_{\left(\mathrm{mm}\right)}}\right)\right]}{2}$$

The length of functional CAD was defined as the length, in millimeters, with µFR drop ≥ 0.0015/mm.

The local physiological disease severity was estimated using the instantaneous µFR gradient per unit of length (dµFR/ds), defined as the maximum µFR gradient in 1 mm. The mean value of dµFR/ds was applied to identify the presence or the absence major gradients.

### 2.7 Physiological Pattern Characterization

Five expert interventional cardiologists specialized in coronary physiology were asked to review and interpret the iFR and µFR pullback traces, in a blind fashion. Operators were instructed to categorize the traces according to previously reported criteria [19, 20]. Focal lesions were defined as an abrupt pressure drop (ΔiFR ≥ 0.03) localized over a relatively short vessel segment (20 mm), whereas the rest of the pullback does not reveal a further significant pressure gradient. Diffuse disease was characterized by a progressive decrease of the pressure gradient in a diseased segment > 20 mm without clearly identifiable focal pressure drops. Mixed patterns of disease were further classified as mixed‐focal or mixed‐diffuse [19].

The 20 mm segment length was chosen based on prior studies that define focal disease as a significant pressure drop occurring within ≤ 20 mm [6, 15, 17], both in hyperemic and non‐hyperemic conditions.

The inter‐operator consensus was used in the present analysis as reference standard. All operators analyzed the iFR Pullback traces in a blinded manner. The vessel was classified based on the most chosen pattern. In case of a tie, classification was determined through a consensus reached via collegial discussion.

Central illustration shows examples of both qualitative and quantitative physiological pattern of CAD.

### 2.8 Statistical Analysis

Continuous variables are presented as mean and standard deviation if normally distributed or with median value and interquartile range otherwise. Continuous variables were compared with unpaired *t*‐test, one way ANOVA or the Mann Whitney test as appropriate. Categorical data are reported as number (percentage) and compared with the χ^2^ test or Fisher exact test as appropriate. Correlation among variables was determined by Pearson correlation tests and expressed as r value. The agreement between physiology indices including iFR, FFR, and µFR was assessed with scatter plots and with the Bland Altman analysis. Sensitivity, specificity, diagnostic accuracy, and optimal cut‐off value were defined from the calculated receiver operator characteristic (ROC) curve. Inter‐rater agreement for visual classification was assessed using Fleiss' kappa (κ). A *p*‐value < 0.05 indicated that the agreement was significantly greater than expected by chance. All analyses were performed with Jamovi® (Version 2.5.7, @jamoviStats Sydney, Australia).

### 2.9 Ethical Considerations

The study was conducted in accordance with the ethical principles of the Declaration of Helsinki and was approved by the local institutional ethics board. Written informed consent was obtained from all patients.

## 3 Results

### 3.1 Clinical and Angiographic Characteristics of the Study Cohort

Overall, 198 patients with 209 coronary vessels were included in the study. The clinical characteristics of the study cohort are reported in Table 1. Briefly, the mean age was 67.7 ± 11.0 SD years and 17.7% of patients were female. The clinical presentation was chronic coronary syndrome in 60.3% and acute coronary syndrome (ACS) in 39.7%. Most of the interrogated vessels showed lesions of intermediate angiographic severity with a mean diameter stenosis of 43.4 ± 8.90 SD%. The mean FFR, iFR, and µFR were 0.79 ± 0.08 SD, 0.83 ± 0.12 SD, and 0.74 ± 0.14 SD, respectively. Correlation between FFR and iFR was r = 0.706.

**TABLE 1:** Baseline clinical characteristics according to disease patterns determined by iFR pullback qualitative longitudinal analysis.

	Total (*n* = 209)	Focal (*n* = 44, 22%)	Mixed focal (*n* = 61, 29%)	Mixed diffuse (*n* = 34, 16%)	Diffuse (*n* = 70, 33%)	*p* value
Demographics						
Age, y	67.7 ± 11.0	68.8 ± 11.9	68.9 ± 11.7	66.4 ± 10.1	66.6 ± 10.3	0.531
Male	172 (82.3)	35 (79.5)	43 (70.5)	28 (82.3)	66 (94.3)	0.005
BMI	26.8 ± 3.78	26.5 ± 3.55	26.4 ± 4.53	27.8 ± 3.67	26.7 ± 3.67	0.715
Risk factors						
Hypertension	156 (74.6)	34 (77.2)	46 (75.4)	24 (70.6)	52 (74.3)	0.923
Hyperlipidaemia	138 (66.0)	27 (61.3)	44 (72.1)	21 (61.7)	46 (65.7)	0.633
Diabetes mellitus	67 (32.1)	12 (27.2)	19 (31.1)	14 (41.1)	22 (31.4)	0.616
Insulin therapy	20 (9.6)	4 (9.1)	6 (9.8)	4 (11.7)	6 (8.5)	0.814
Current smoking	51 (24.4)	11 (25.0)	16 (26.2)	4 (11.7)	20 (28.5)	**0.010**
Chronic kidney disease	53 (25.4)	14 (31.8)	17 (27.8)	8 (23.5)	14 (20.0)	0.571
Dialysis	6 (2.9)	1 (2.2)	2 (3.2)	1 (2.9)	2 (2.8)	0.632
PAD	67 (32.1)	14 (31.8)	19 (31.1)	9 (26.4)	25 (35.7)	0.815
Previous PCI	62 (29.6)	10 (22.7)	18 (29.5)	12 (35.3)	22 (31.4)	0.651
Previous CABG	2 (1.0)	1 (2.2)	1 (1.6)	0 (0.0)	0 (0.0)	0.550
Clinical presentation						
ACS	83 (39.7)	18 (40.9)	28 (45.9)	11 (32.3)	26 (37.1)	0.580
STEMI	26 (12.4)	8 (18.1)	7 (11.4)	4 (11.7)	7 (10.0)	0.686
Culprit lesion functionally investigated	24 (11.5)	3 (6.8)	7 (11.4)	4 (11.7)	10 (14.3)	0.686
Stable CAD	126 (60.3)	26 (59.1)	33 (54.1)	23 (67.6)	44 (62.8)	0.580

Abbreviations: ACS, acute coronary syndrome; BMI, body mass index; CABG, coronary artery bypass graft; CAD, coronary artery disease; PAD, peripheral artery disease; PCI, percutaneous coronary intervention; STEMI, ST‐Elevation myocardial infarction.

### 3.2 iFR‐Based Qualitative Vessel Longitudinal Analysis

**FIGURE 3:**
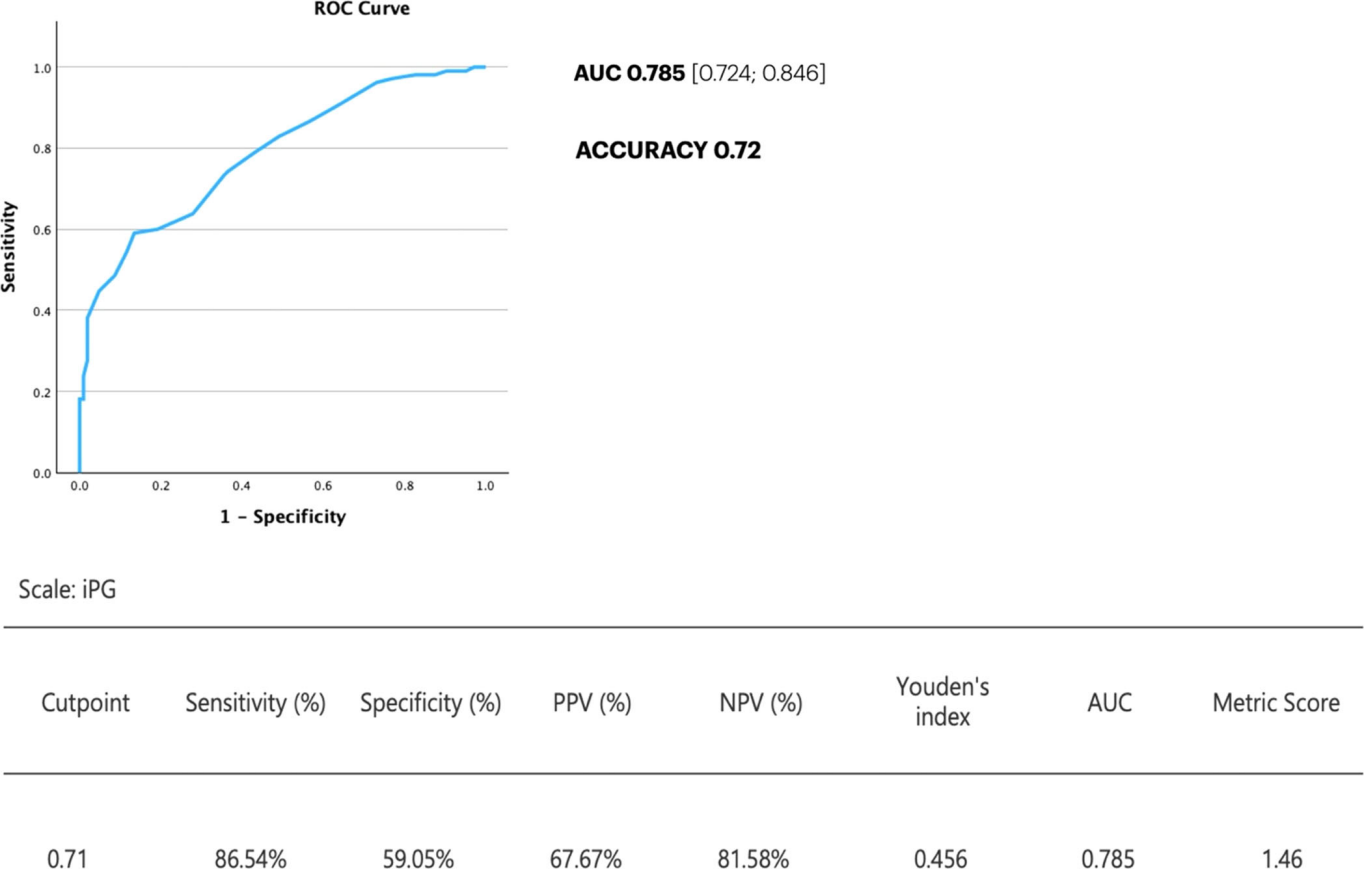
Diagnostic performance of iPG in predicting the physiological pattern of disease. AUC, area under the curve; iPG, instantaneous free‐ratio pullback pressure gradient index; NPV, negative predictive value; PPV, positive predictive value; ROC, receiver operating characteristic. [Color figure can be viewed at wileyonlinelibrary.com]

**FIGURE 4:**
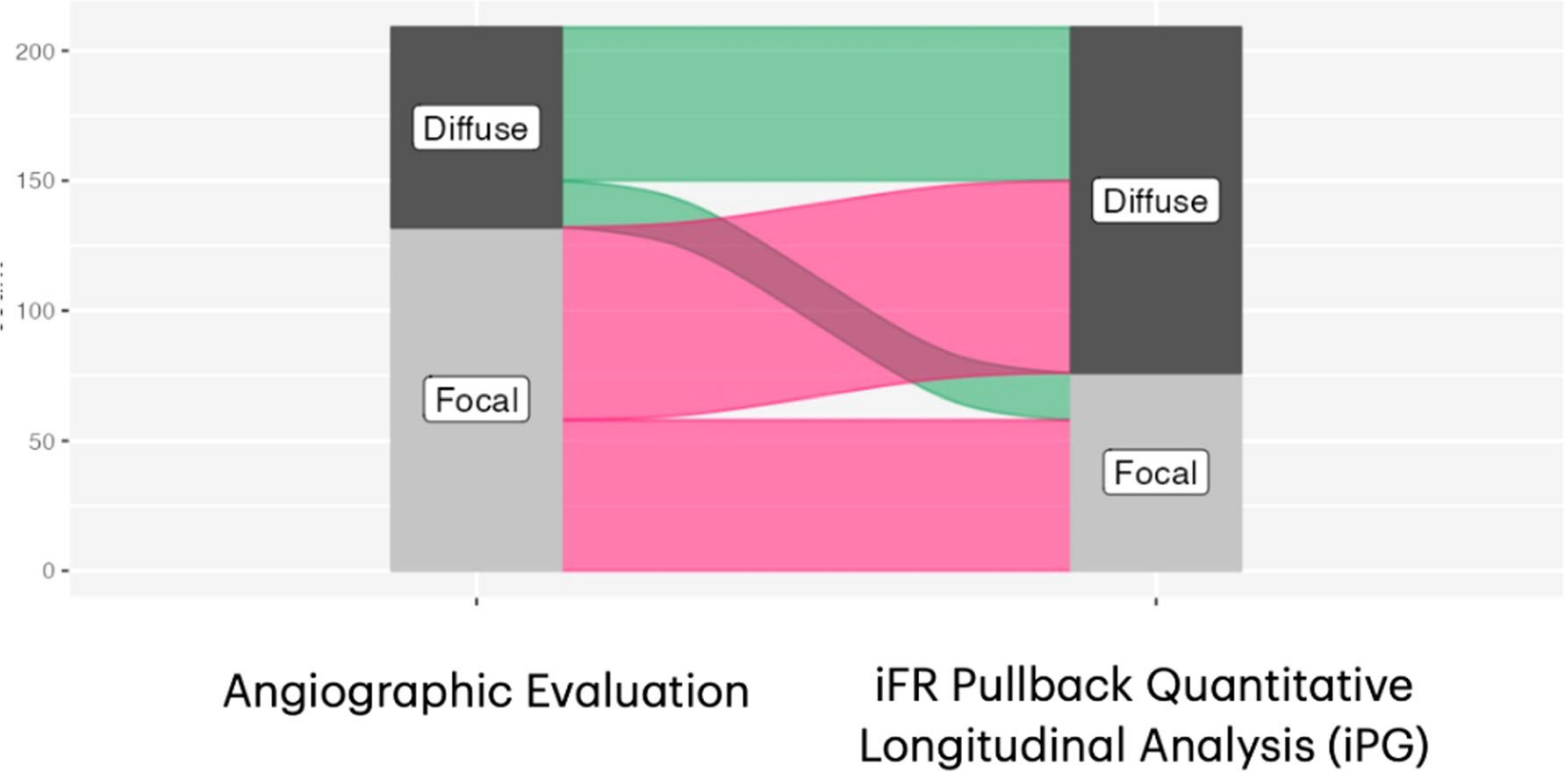
Reclassification of the angiographic patterns based on the quantitative iFR pullback evaluation (iPG). Reclassification of disease patterns at the alluvional plot. 8.6% of vessels classified as diffuse at the angiographic evaluation, was re‐evaluated as focal at the quantitative longitudinal analysis. 35.4% of vessel classified as focal at the angiographic analysis had an iPG indicative of diffuse disease. iFR, instantaneous wave‐free ratio; iPG, Instantaneous free‐ratio pullback pressure gradient index. [Color figure can be viewed at wileyonlinelibrary.com]

At the iFR qualitative vessel longitudinal analysis, experts classified 105 vessels (51%) as predominantly focal (focal and mixed‐focal) and 104 vessels (49%) as predominantly diffuse (diffuse and mixed‐diffuse) (Figure 1, Table 2). Mixed patterns were identified in 95 vessels (45%) (Supporting Information S1: Figure 3). The inter‐operator agreement for iFR pullback visual interpretation was moderate (K Fleiss 0.480, *p* < 0.001). Each operator independently and in a blinded manner classified the vessels into focal, mixed‐focal, mixed‐diffuse, and diffuse patterns. Of the 209 iFR pullback traces analyzed, 196 (93.8%) showed sufficient agreement (≥ 3 out of five reviewers with the same classification), while 13 (6.2%) required collegial discussion to achieve consensus (Supporting Information S1: Figure 4). For binary analyses, when necessary, mixed‐focal was grouped with focal, and mixed‐diffuse was grouped with diffuse. In analyses using three categories, mixed‐focal and mixed‐diffuse were combined into a single “mixed” category.

**TABLE 2:** Angiographic characteristics according to disease patterns determined by iFR pullback qualitative longitudinal analysis.

	Total (*n* = 209)	Focal (*n* = 44, 22%)	Mixed focal (*n* = 61, 29%)	Mixed diffuse (*n* = 34, 16%)	Diffuse (*n* = 70, 33%)	*p* value
Target vessel						**< 0.001**
Left Main	4 (1.9)	0 (0.0)	1 (1.6)	0 (0.0)	3 (4.2)	
LAD	187 (89.5)	32 (72.7)	56 (91.8)	33 (97.0)	66 (94.2)	
LCX	12 (5.7)	8 (18.1)	3 (4.9)	0 (0.0)	1 (1.4)	
RCA	6 (2.9)	4 (9.1)	1 (1.6)	1 (2.9)	0 (0.0)	
Quantitative coronary analysis						
RVD	2.77 ± 0.52	2.74 ± 0.48	2.86 ± 0.55	2.75 ± 0.45	2.72 ± 0.54	0.514
DS%	43.4 ± 8.90	47.66 ± 9.53	43.52 ± 8.54	43.16 ± 8.61	40.64 ± 7.99	**0.001**
Physiological indices						
FFR	0.78 ± 0.07	0.77 ± 0.11	0.77 ± 0.07	0.77 ± 0.06	0.80 ± 0.05	**0.022**
iFR	0.83 ± 0.12	0.79 ± 0.178	0.81 ± 0.13	0.84 ± 0.07	0.80 ± 0.08	**0.016**
µFR	0.73 ± 0.14	0.73 ± 0.15	0.73 ± 0.13	0.77 ± 0.13	0.72 ± 0.13	**0.011**
iPG	0.69 ± 0.07	0.78 ± 0.08	0.70 ± 0.08	0.65 ± 0.06	0.66 ± 0.04	**0.002**
diFR/ds	0.023 ± 0.028	0.039 ± 0.047	0.029 ± 0.029	0.017 ± 0.007	0.021 ± 0.024	**0.020**
µFR PPGi	0.66 ± 0.09	0.70 ± 0.11	0.65 ± 0.08	0.69 ± 0.08	0.65 ± 0.08	**0.019**
dµFR/ds	0.030 ± 0.029	0.045 ± 0.049	0.028 ± 0.021	0.029 ± 0.020	0.022 ± 0.018	**0.022**

*Note:* Numbers are count (percentage) or mean (±standard deviation) diFR/ds, instantaneous iFR gradients per unit of length; dµFR/ds, instantaneous µFR gradients per unit of length; DS, diameter stenosis; FFR, fractional flow reserve; iFR, instantaneous wave‐free ratio; iPG, instantaneous free‐ratio pullback pressure gradient index; LAD, left anterior descending artery; LCX, left circumflex artery; µFR, Murray's law quantitative flow ratio; µFR PPGi, µFR pullback pressure gradient index; RCA, right coronary artery; RVD, reference vessel diameter.

### 3.3 iFR‐Based Quantitative Vessel Longitudinal Analysis

The mean value of iPG was 0.697. iPG was significantly higher in vessels classified by experts as focal (iPG: 0.786 ± 0.081 SD) compared to vessels with mixed‐focal (0.702 ± 0.058 SD), mixed‐diffuse (0.650 ± 0.066 SD), or diffuse pattern (0.661 ± 0.048 SD) (*p* < 0.0001 for all comparisons) (Figure 2, Table 2). Similarly, the diFR/ds was significantly higher in vessels with focal (0.0391 ± 0.0470 SD) compared with vessels with mixed‐focal (0.0290 ± 0.0292 SD), mixed‐diffuse (0.0176 ± 0.0078 SD) or diffuse pattern (0.0129 ± 0.0072 SD) (*p* < 0.0001 for all comparisons) (Figure 2, Table 2). iPG demonstrated an overall good accuracy in predicting the predominant pattern of disease, based on iFR‐pullback qualitative interpretation (AUC 0.785, CI 0.95: 0.724–0.846; *p* < 0.001, Figure 3). iPG reclassified 44% of the patterns compared with the qualitative angiographic interpretation (Figure 4). The mean value of diFR/ds (0.0239 ± 0.0289 SD) in the present population was used to define the presence or absence of major pressure drops. Patients with major drops showed higher mean iPG values compared to those without, although this difference was not statistically significant (0.716 ± 0.0987 SD vs 0.692 ± 0.0725 SD; *p* = 0.067) (Supporting Information S1: Figure 5).

### 3.4 µFR‐Based Qualitative Vessel Longitudinal Analysis

Based on µFR virtual pullback, 102 (49%) vessels were classified as predominantly focal and 107 (51%) as predominantly diffuse (Figure 1). The overall agreement with the iFR based pullback analysis was 60.3% (k = 0.206, *p* = 0.003, Supporting Information S1: Figure 6).

Mixed patterns were observed in 81 (39%) vessels (Supporting Information S1: Figure 7).

The inter‐operator agreement on µFR virtual pullback was moderate (K Fleiss 0.440). Of the 209 µFR pullback traces analyzed, 200 (95.7%) showed sufficient agreement (≥ 3 out of five reviewers with the same qualitative classification), while 10 (4.3%) required collegial discussion to achieve consensus (Supporting Information S1: Figure 4).

### 3.5 µFR‐Based Quantitative Vessel Longitudinal Analysis

The mean value of µFR‐derived PPGi was 0.663. µFR‐derived PPGi was significantly higher in vessels with focal lesions (0.776 ± 0.060 SD) compared with vessels with diffuse patterns (0.627 ± 0.084 SD) or mixed patterns, both mixed‐focal (0.660 ± 0.066 SD) and mixed‐diffuse (0.595 ± 0.071 SD). Also, the dµFR/ds was significantly higher in vessels with focal disease (0.051 ± 0.048 SD) with respect to vessels with mixed‐focal (0.037 ± 0.024 SD), mixed‐diffuse (0.024 ± 0.017 SD) or diffuse disease (0.015 ± 0.007 SD) (*p* < 0.0001) (Table 2, Supporting Information S1: Figure 8 and Supporting Information S1: Table 1).

The µFR‐derived PPGi demonstrated an overall good accuracy in predicting the predominant pattern of disease (AUC 0.77, CI 0.95, *p* < 0.001). The best threshold of µFR‐derived PPGi to define focal disease was 0.66 (Youden index = 0.426) providing a sensitivity, specificity, positive predictive value, negative predictive value and accuracy of 71.96%, 70.59%, 71.96%, 70.59% and 0.71 respectively (Supporting Information S1: Figure 9).

Based on µFR‐derived PPGi, 102 (49%) vessels were classified as predominantly focal and 107 (51%) as predominantly diffuse (Figure 1).

### 3.6 Comparison of µFR‐ and iFR‐Based Quantitative Vessel Longitudinal Analysis

µFR‐derived PPGi demonstrated a modest, but significant correlation with iPG (r = 0.238, *p* < 0.001). The Bland Altman analysis showed a bias of 0.03 and it is reported in Supplementary Figure 10. dµFR/ds demonstrated a moderate significant correlation with diFR/ds (r = 0.528; *p* < 0.001). The Bland Altman analysis showed a bias of −0.006.

## 4 Discussion

This study introduces iPG and diFR/ds as novel, non‐hyperemic physiological indices that provide complementary insights into the distribution and severity of coronary artery disease.

The iPG offers a continuous, vessel‐level metric that reflects the overall physiological distribution of disease, while diFR/ds quantifies local pressure gradients, highlighting major focal drops along the vessel. Their combined use enables a more refined, non‐hyperemic assessment of coronary lesions, capturing both diffuse and focal components.

These findings suggest that iPG and diFR/ds may enhance physiological interpretation of coronary disease beyond binary significance thresholds, supporting a more comprehensive evaluation of intermediate lesions in real‐world practice.

iPG and diFR/ds provide complementary insights into coronary physiology, as they assess different aspects of vascular disease. Specifically:

- iPG offers a global assessment of the overall disease burden in the vessel, reflecting the total physiological impact of stenoses.
- ΔiFR/ds, on the other hand, provides a spatially resolved measure of pressure gradients along the vessel, capturing how pressure changes across different segments.

For example, a vessel with diffuse disease might still exhibit localized regions of higher pressure gradients. In such cases, ΔiFR/ds could help identify focal segments within a diffusely diseased artery that may serve as potential targets for revascularization, even when the global iPG value alone does not clearly indicate a specific intervention site.

The main findings of the present study are as follows:

1. The visual interpretation of the pressure‐wire pullback is flawed by significant interobserver variability (K Fleiss 0.440).
2. The iPG and diFR/ds are practical tools for characterizing physiological CAD patterns obviating the need for steady‐state hyperemia. An iPG cut‐off value of 0.71 accurately differentiates between focal and diffuse patterns of CAD (AUC 0.785; *p* < 0.05).
3. Using the ROC derived cut‐off of 0.71, the iPG reclassified 44% of the patterns compared with the angiographic visual interpretation.
4. iPG and diFR/ds provided a moderate agreement with μFR‐derived metrics including μFR‐PPGi and dμFR/ds.

Diffuse disease poses several challenges in patients undergoing PCI including long coronary segments requiring treatment, a lack of adequate landing zone for stenting and impairment of coronary flow related to the diffuse atherosclerotic burden along the vessel [19, 21, 22]. Moreover, diffuse disease is associated with higher risk of suboptimal functional results and residual symptoms after PCI [23, 24, 25]. Therefore, determining the distribution pattern of disease, with the identification of focal lesions versus segments of diffuse disease, has become one of the cornerstones of decision‐making for myocardial revascularization.

The global use of wire‐based physiological assessments in clinical practice remains low and inconsistent. Some reasons for this low adoption include longer procedure times, the invasive nature of the instruments, and the use of vasodilator agents, which can cause undesirable side effects [26]. The non‐hyperemic nature of iPG is especially advantageous, given that traditional hyperemic methods, such as FFR, require the use of adenosine, which can be time‐consuming and uncomfortable for patients.

Our study confirmed that the qualitative interpretation of iFR pullbacks is hampered by significant interobserver variability, emphasizing the need for standardization in clinical practice. Similar to what has been observed for FFR‐based PPGi, the higher the iPG, the more focal the lesion, while lower iPG values indicate more diffuse disease [3, 5]. This finding mirrors the results obtained from μFR‐PPGi, a purely angiogram‐based method that also demonstrated higher values in focal lesions [19]. In our analysis, iPG and μFR‐PPGi were significantly correlated. Moreover, iPG also correlated with local lesion severity, as evidenced by its relationship with diFR/ds (r = 0.528, *p* < 0.001).

The combination of iPG and diFR/ds provides complementary insights into the overall and localized physiological impact of CAD, offering clinicians a robust set of tools to guide revascularization strategies.

The potential clinical impact of iPG is significant, particularly in light of its ability to reclassify lesions previously deemed focal based on visual angiographic assessment. Notably, iPG reclassified 35.4% of vessels initially interpreted as focal, suggesting that relying solely on qualitative analysis may lead to misclassification, with potential consequences for treatment decisions. Therefore, the integration of quantitative tools such as iPG into routine clinical practice could reduce subjectivity and improve decision‐making accuracy in PCI planning.

﻿A key finding of our study is that mixed patterns are both common (~45% of cases) and often underrecognized when dichotomous variables, such as the PPGi, are used. In this context, coregistered techniques can be instrumental in accurately identifying focal pressure drops that may be amenable to treatment with PCI. Careful evaluation of both the length and magnitude of the focal drop is essential to predict the anticipated physiological benefit following PCI. Notably, in cases of mixed patterns, PCI tends to yield less favorable physiological outcomes compared to scenarios with isolated focal lesions, due to the concurrent presence of diffuse disease [3, 23, 24, 27]. From a procedural standpoint, in patients with a functional mixed pattern, as for diffuse disease, intracoronary imaging should be utilized to identify a disease‐free landing zone for stenting. This approach helps optimize post‐PCI outcomes by allowing detailed assessment of stent edges, which is crucial given the heightened risk of complications such as dissections or plaque shift associated with diffusely diseased segments.

Future research could explore several avenues. Prospective studies should evaluate whether the use of iPG can improve clinical outcomes by influencing PCI strategies.

Finally, efforts should be made to improve the inter‐operator reliability of qualitative assessments, possibly through advanced machine learning algorithms that can standardize interpretation. The ongoing DEFINE GPS (NCT04451044) study will contribute to a comprehensive understanding of how invasive physiological assessments with iFR can optimize PCI compared to angiography alone in terms of clinical outcomes.

### 4.1 Limitations

This study has several limitations. First, selection bias may have occurred due to its observational, single‐center design. However, efforts were made to minimize confounding factors, such as excluding angiograms and pullback traces with poor quality; all analyses were conducted by experienced operators. Low‐quality angiographic analyses and pullback traces could have negatively impacted the reproducibility of µFR assessments and the accuracy of pullback graphical data extraction.

Second, iFR pullback traces do not allow precise determination of vessel length, as the curves are plotted with time on the x‐axis. To address this, vessel length extrapolated from the µFR analysis was used for both iFR and virtual µFR pullback data extractions.

Third, manual iFR pullback may have influenced trace shape. Nonetheless, all procedures were performed by experienced operators instructed to maintain pullback velocities between 0.5 and 1.0 mm/s. Avoiding motorized pullback may even reflect real‐world clinical practice. Furthermore, previous studies have demonstrated that manual pullbacks offer comparable accuracy to motorized systems, with validated inter‐operator reproducibility and strong clinical relevance [28].

Fourth, the cohort predominantly included LAD vessels (~90%), which may limit generalizability. Future studies with a more balanced vessel distribution are warranted.

Lastly, iPG and diFR/ds were not compared with hyperemic counterparts. However, since µFR‐based PPGi and FFR‐based PPGi are correlated, µFR PPGi was used as a surrogate in this analysis to preserve physiological relevance. Central Illustration 1.

**Central Illustration 1:**
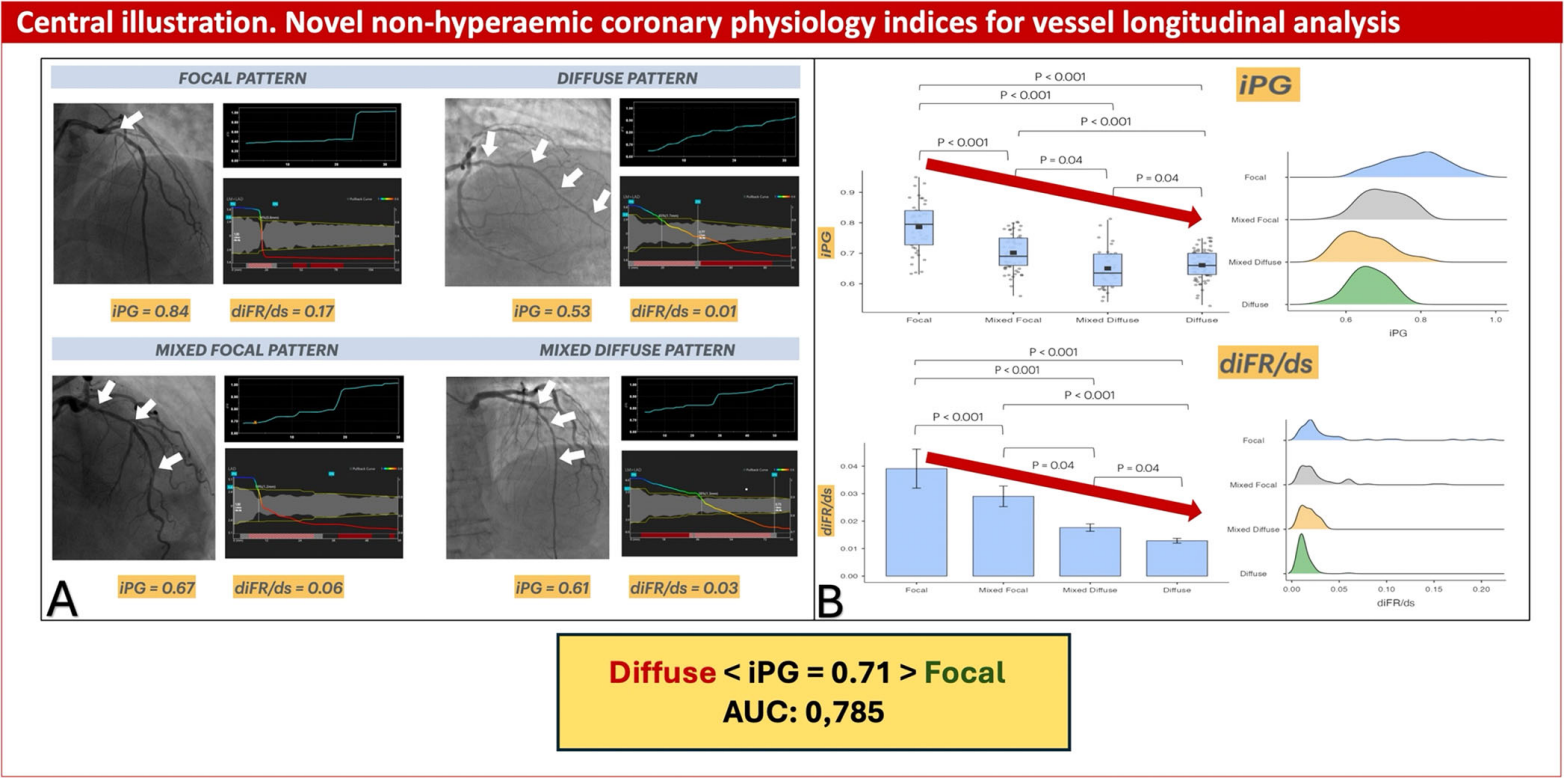
Novel non‐hyperemic coronary physiology indices for vessel longitudinal analysis. Novel coronary physiology indices, iPG and diFR/ds, characterize the physiological patterns of coronary vessels and local disease severity without the need of hyperemia. In panel A, an illustrative case of each type of disease is given. For each case, angiography (pointing out the presence of disease), iFR pullback, µFR pullback, iPG and diFR/ds values are reported. In the upper part of panel B, iPG values are reported in reference to the disease pattern in form of box plot (on the left) and density plot (on the right). In the lower part of panel B, diFR/ds values are reported in reference to the disease pattern in form of bar chart (on the left) and density plot (on the right). Higher iPG and diFR/ds values are associated with focal disease, while lower values of iPG and diFR/ds are indicative of diffuse disease. diFR/ds, Instantaneous iFR gradients per unit of length; iFR, instantaneous wave‐free ratio; iPG, Instantaneous free‐ratio pullback pressure gradient index; µFR, Murray's law quantitative flow ratio. [Color figure can be viewed at wileyonlinelibrary.com]

## 5 Conclusion

The integration of novel non‐hyperemic indices (iPG and diFR/ds) enhances the physiological interpretation of coronary artery disease by enabling longitudinal vessel and lesion‐level analysis. iPG, based on resting physiology, offers a simple and user‐friendly alternative to PPGi without requiring steady‐state hyperemia. These indices provide objective, reproducible metrics, reducing interobserver variability and improving lesion classification, especially in distinguishing focal from diffuse patterns. The ability of iPG to reclassify nearly 30% of lesions previously considered focal underscores its value in refining atherosclerotic disease assessment. Incorporating iPG and diFR/ds into clinical practice may optimize treatment strategies through more accurate lesion characterization, identification of disease‐free stent landing zones, and improved PCI planning and outcomes.

## Consent

All patients provided written informed consent for inclusion in the study and for the use of anonymized data for research purposes.

## Ethics Statement

The study was conducted in accordance with the Declaration of Helsinki. Ethical approval was obtained from the local institutional ethics board.

## Conflicts of Interest

R. Scarsini has received speaker and consultation fees from Abbott and B‐Braun, and institutional research grants from Edwards Lifesciences, Abbott and Medis. S. Fezzi has received consultancy fees from Boston Scientific and Biotronik. S. Tu is a cofounder of Pulse Medical and reports research grant and consultancy from Pulse Medical. Dr. Prado has been supported by a research grant provided by the DigiCardiopaTh PhD Program. All other authors declare that they have no conflicts of interest related to this study.

## Supporting information

Supplementary Data.

## Data Availability

The data that support the findings of this study are not publicly available due to their potential use in future related publications. Reasonable requests for access may be considered by the corresponding author.
