# Supplementary material for: Novel Non‐Hyperemic Coronary Physiology Indices for Vessel Longitudinal Analysis
Source: Catheter Cardiovasc Interv. 2025 Aug 5;106(4):2374–85. doi: 10.1002/ccd.70065 (PMC12502034; doi:10.1002/ccd.70065)

**Supplementary Data**

**Novel non-hyperemic coronary physiology indices for vessel longitudinal analysis.**

Simone Fezzi MD Msc^1^, Guy F A Prado MD^1-2^, Luigi Alberto Iossa MD^1^, Daixin Ding Msc PhD^3^, Elisabetta Pianezzola MD^1^, Federico Cesar Vigo MD^1^, Paolo Alberto Del Sole MD^1^, Verdiana Galli MD^1^, Stefano Andreaggi MD^1^, Domenico Tavella MD^1^, Shengxian Tu MD PhD^3^, Gabriele Pesarini MD PhD^1^, Flavio Ribichini MD^1^, Roberto Scarsini MD PhD^1^

^1^Division of Cardiology, Department of Medicine, University of Verona, Piazzale A. Stefani 1, 37126 Verona, Italy

^2^Department of Clinical and Molecular Medicine, Sapienza University, Viale Regina Elena 324, 00161 Rome, Italy

^3^Department of Cardiology, Ren Ji Hospital, School of Medicine and School of Biomedical Engineering, Shanghai Jiao Tong University, 160 Pujian Road, Shanghai 200127, China

**Table of Contents**

- **Supplementary Table**
- **Supplementary Figures**

**Supplementary Table and Figures**

**Supplementary Table 1. Mean, minimum and maximum µFR PPGi values for each kind of pattern disease.**

µFR PPGi, µFR pullback pressure gradient index.

| **μFR Qualitative Longitudinal Analysis** | **N** | **Mean** | **SD** | **Minimum** | **Maximum** |
| --- | --- | --- | --- | --- | --- |
| **Focal** | 43 | 0.776 | 0.0606 | 0.680 | 0.920 |
| **Predominantly Focal** | 59 | 0.660 | 0.0665 | 0.500 | 0.790 |
| **Predominantly Diffuse** | 22 | 0.595 | 0.0713 | 0.490 | 0.790 |
| **Diffuse** | 85 | 0.627 | 0.0848 | 0.400 | 0.850 |

**Supplementary Figure 1. Fluoroscopic wire tip position and corresponding µFR distal landmark.**

Coronary angiography of the LAD, showing the position of the distal pressure-wire sensor, highlighted by the red arrow (Panel A). Corresponding µFR anatomical reconstruction of the LAD, with the red arrow indicating the distal reference point (“distal normal” segment) used for vessel length standardization (Panel B). This anatomical point was carefully matched to the pressure-wire tip location identified in Panel A.

LAD = left anterior descending artery; µFR, Murray’s law quantitative flow ratio.


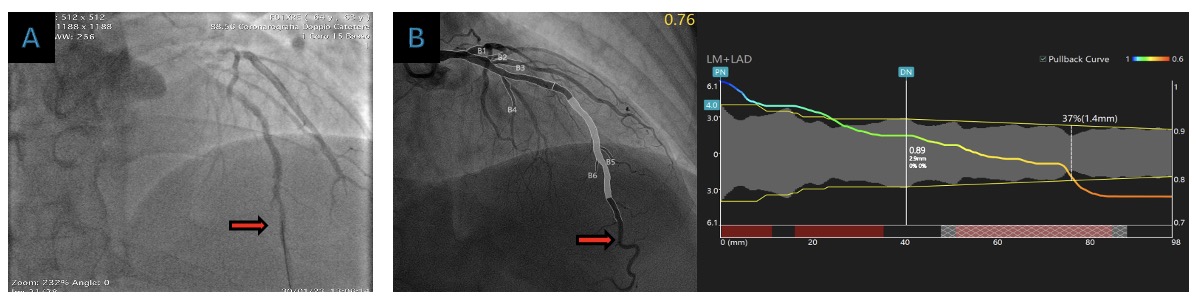


**Supplementary Figure 2. Numerical data extraction from both the iFR pullback and the µFR virtual pullback using WebPlotDigitizer.** The graphs were calibrated by setting reference points on the x- and y-axes (Panel A). To enhance accuracy, the 'Pen' tool was used to restrict the recognition area, ensuring precise extraction of relevant data points (Panel B). Then, data points were automatically digitized using the built-in detection algorithm (Panel C). Extracted values were then exported for further statistical analysis.

iFR, Instantaneous Flow Ratio; µFR, Murray’s law quantitative flow ratio.

**
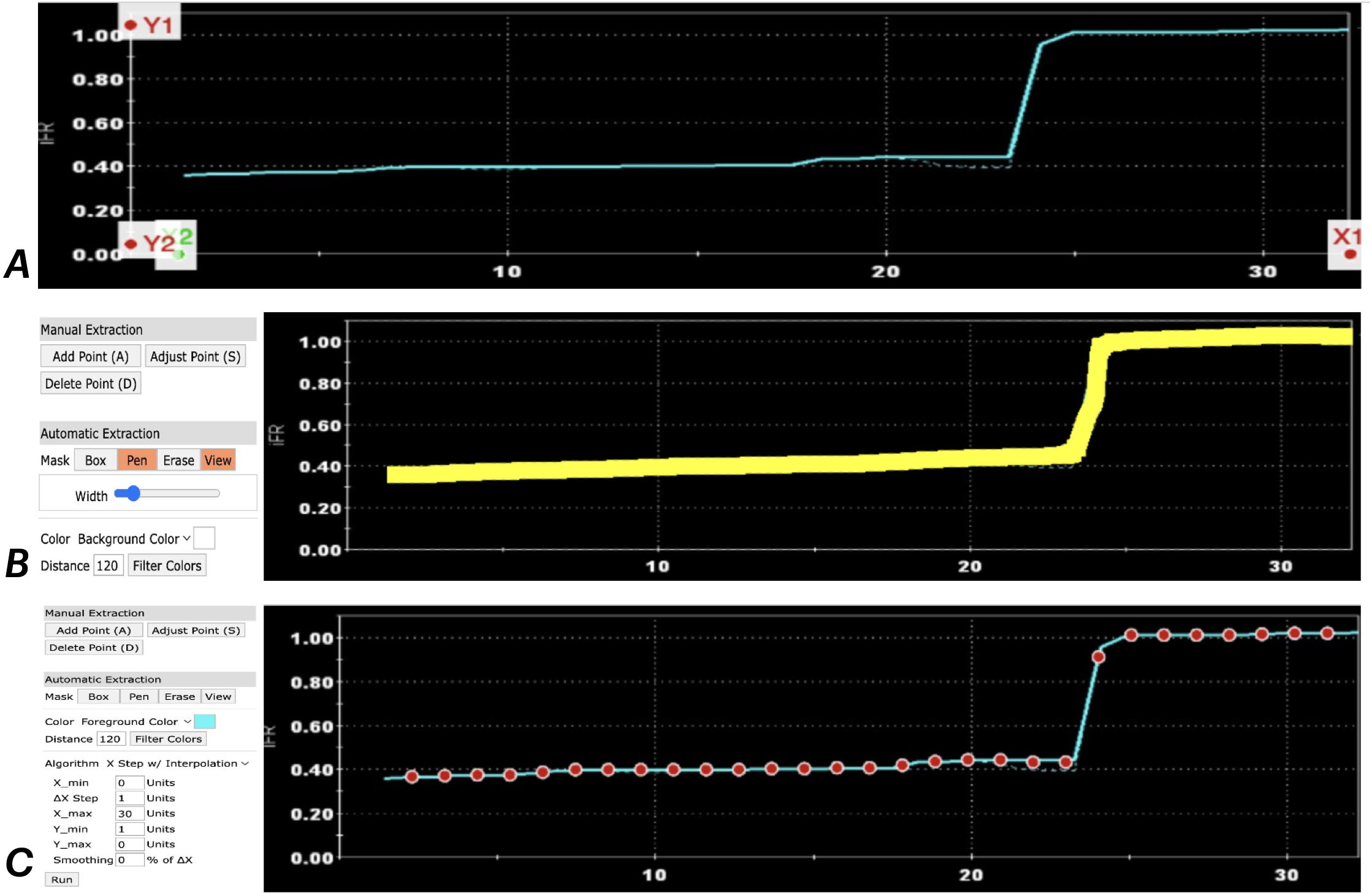
**

**Supplementary Figure 3. Proportion of vessels distinct into focal, mixed focal, mixed diffuse or diffuse pattern at qualitative longitudinal analysis of the iFR Pullback.**

iFR, Instantaneous Flow Ratio.


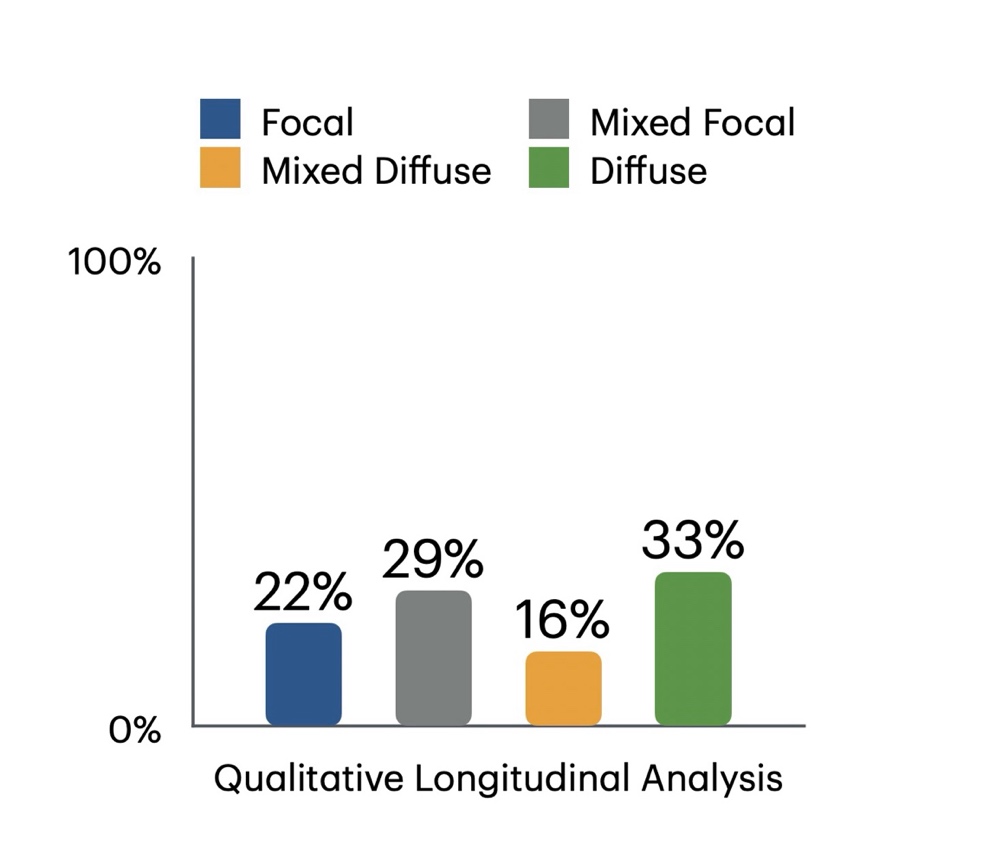


**Supplementary Figure 4. Inter-Observer Agreement for iFR and µFR Pullback Pattern Interpretation.**

Pie charts illustrating the level of consensus among five independent reviewers in classifying the physiological pattern (focal, diffuse, predominantly focal, or predominantly diffuse) of each vessel based on iFR (left panel) and µFR (right panel) pullback traces.
"Consensus (≥3/5 reviewers)" was achieved in 196 out of 209 cases (93.8%) for iFR and 200 out of 209 cases (95.7%) for µFR.
In the remaining cases (6.2% for iFR, 4.3% for µFR), collegial discussion was required to reach a final classification.


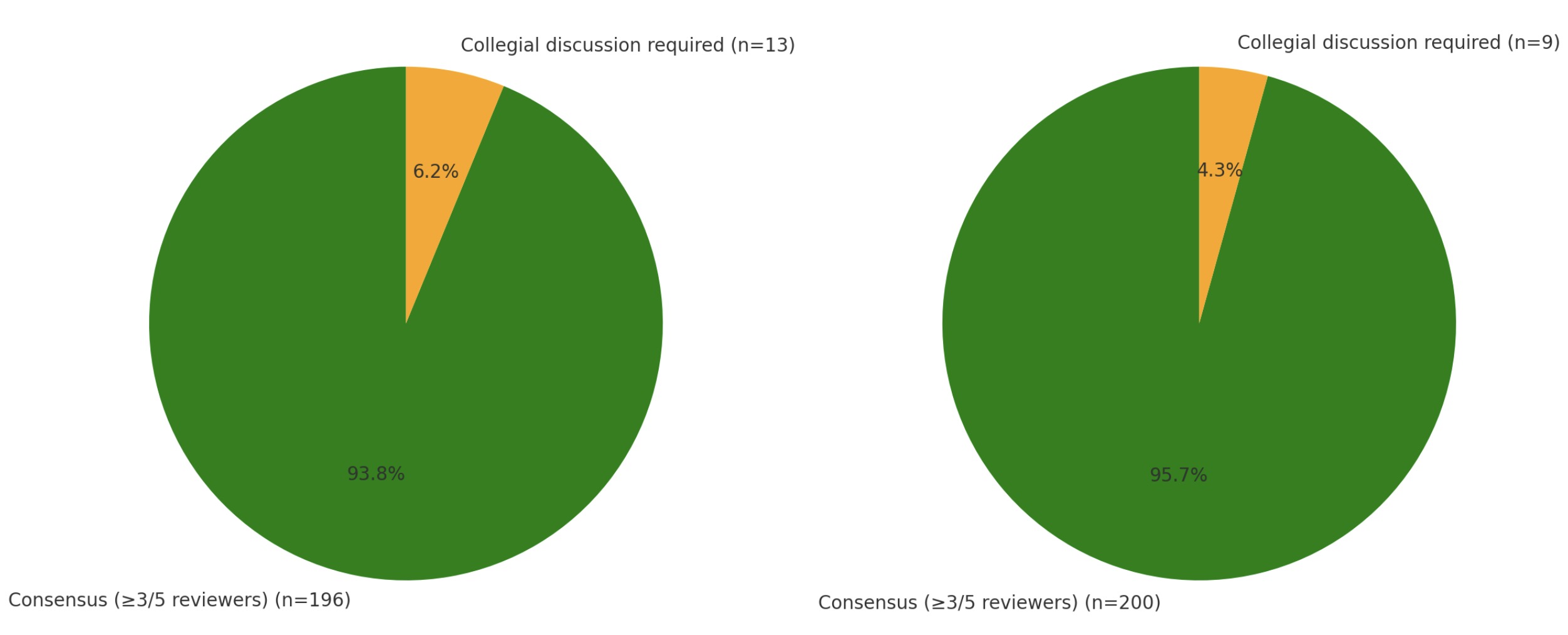

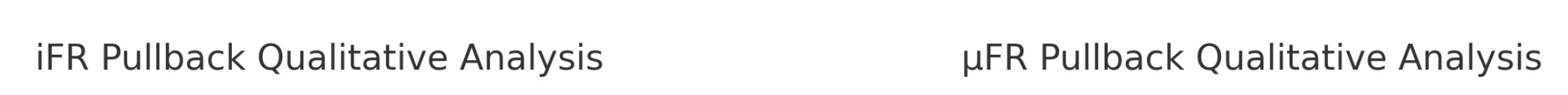


**Supplementary Figure 5. iPG distribution with respect to the presence of major drops. Vessels with major drops (diFR/ds>0.0239) have, on average, higher iPG values.**

iPG, iFR Pressure Gradient.


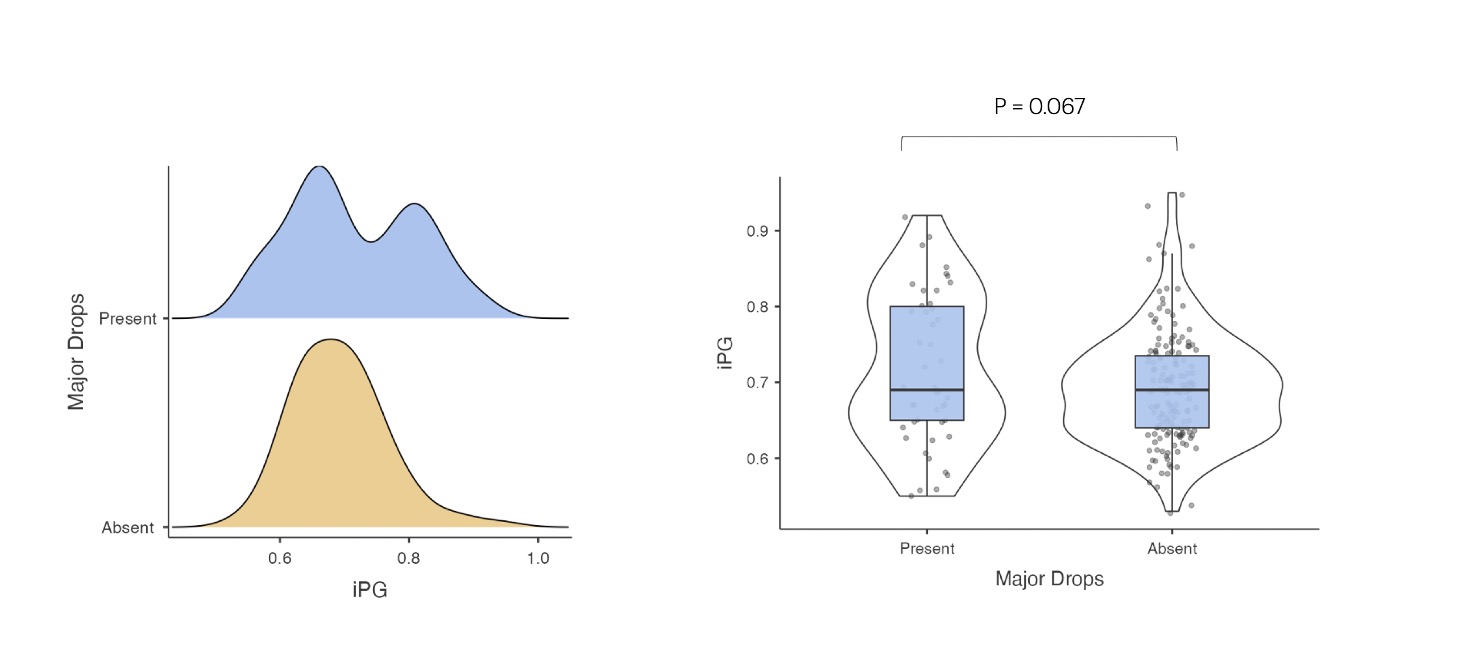


**Supplementary Figure 6. Correspondence between µFR Qualitative Longitudinal Analysis and iFR Qualitative Longitudinal Analysis.**

On the X-Axis there is the µFR qualitative evaluation. The different colors in the columns represent the iFR qualitative evaluation. The 41% of vessels identified at µFR evaluation as focal, was identified as focal also at iFR analysis. The 52% of vessels identified at µFR evaluation as mixed, was identified also as mixed at iFR analysis. The 46% of vessels identified at µFR evaluation as diffuse, was identified as diffuse at iFR analysis.

iFR, Instantaneous Flow Ratio; µFR, Murray’s law quantitative flow ratio.


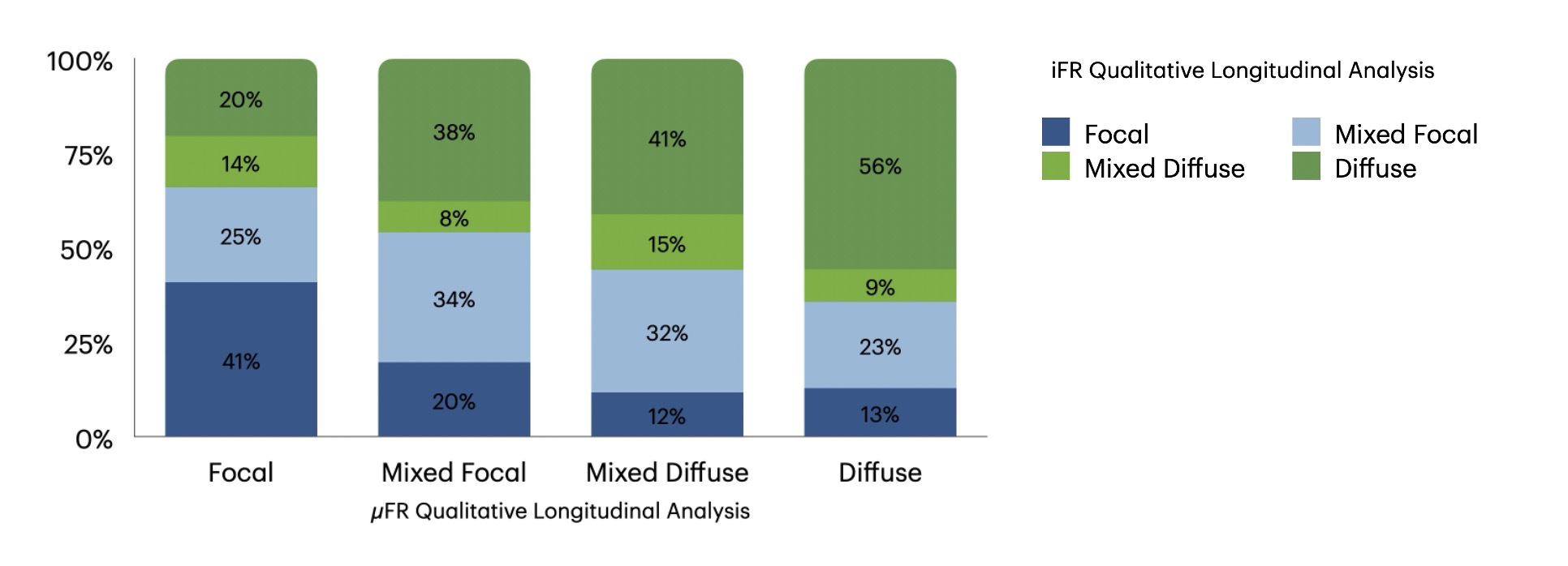


**Supplementary Figure 7. Distribution of vessels at µFR qualitative longitudinal analysis differentiating between focal and diffuse disease (Panel A) and focal, diffuse, mixed focal and mixed diffuse disease (Panel B).**

µFR, Murray’s law quantitative flow ratio.


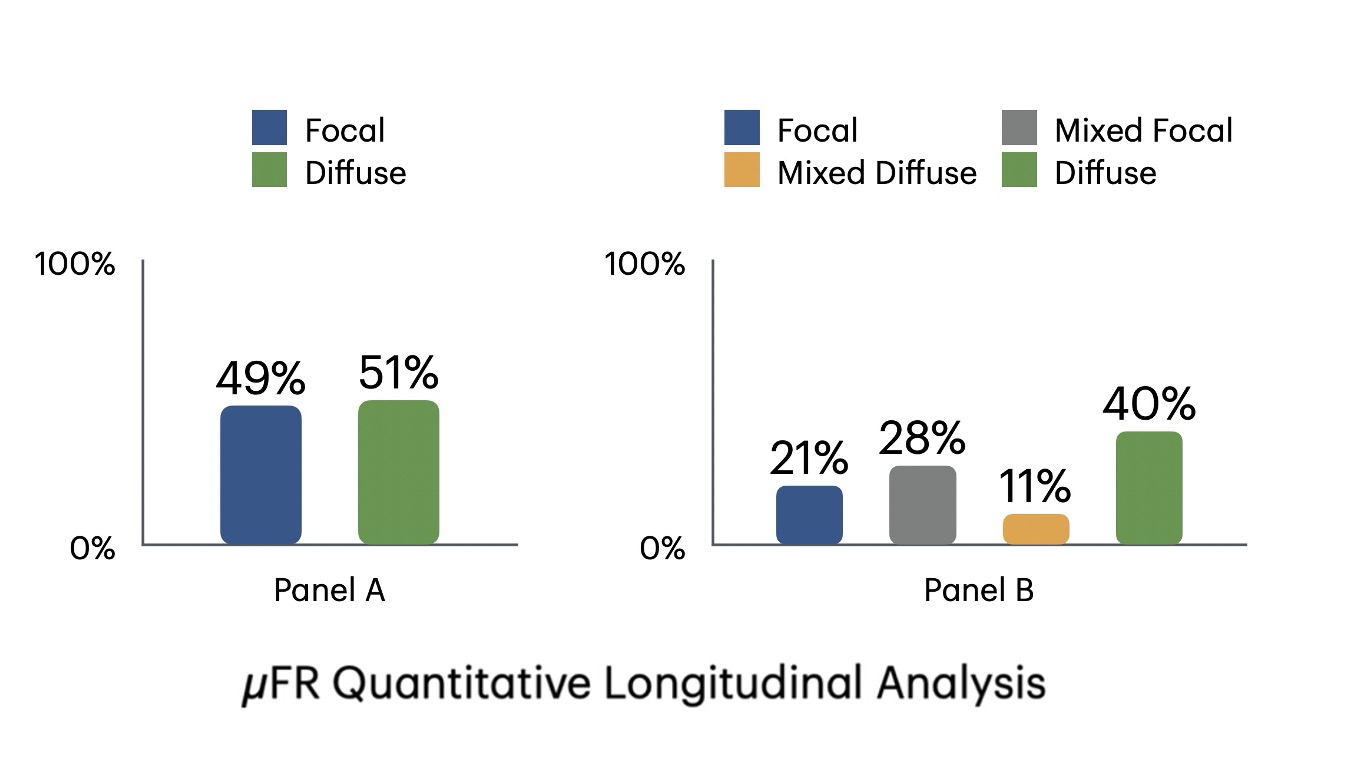


**Supplementary Figure 8. Distribution of µFR PPGi and dµFR/ds values with respect to the type of disease assessed at the µFR qualitative longitudinal analysis.** In the upper part, µFR PPGi data are represented through box plot (on the left) and density plot (on the right). In the lower part dµFR/ds values distribution is represented in a bar chart (on the left) and in a density plot (on the right).

dµFR/ds, Instantaneous µFR gradients per unit of length; µFR, Murray’s law quantitative flow ratio; µFR PPGi, µFR pullback pressure gradient index.**
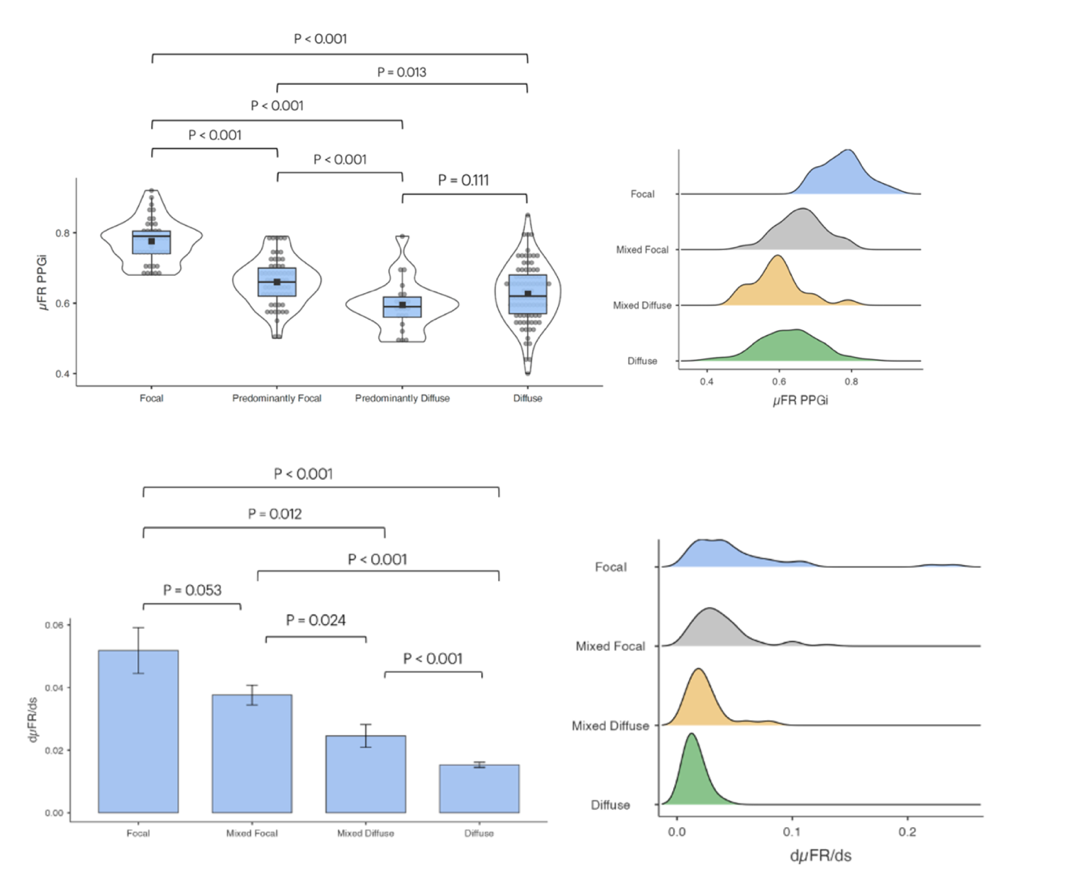
**

**Supplementary Figure 9. ROC curves analysis. Diagnostic performance of µFR PPGi in predicting the disease pattern in the overall population and according to µFR pullback qualitative longitudinal analysis.**

AUC, Area under the curve; NPV, Negative predictive value; PPV, positive predictive value; ROC, Receiver Operating Characteristic; µFR, Murray’s law quantitative flow ratio; µFR PPGi, µFR pullback pressure gradient index.

***
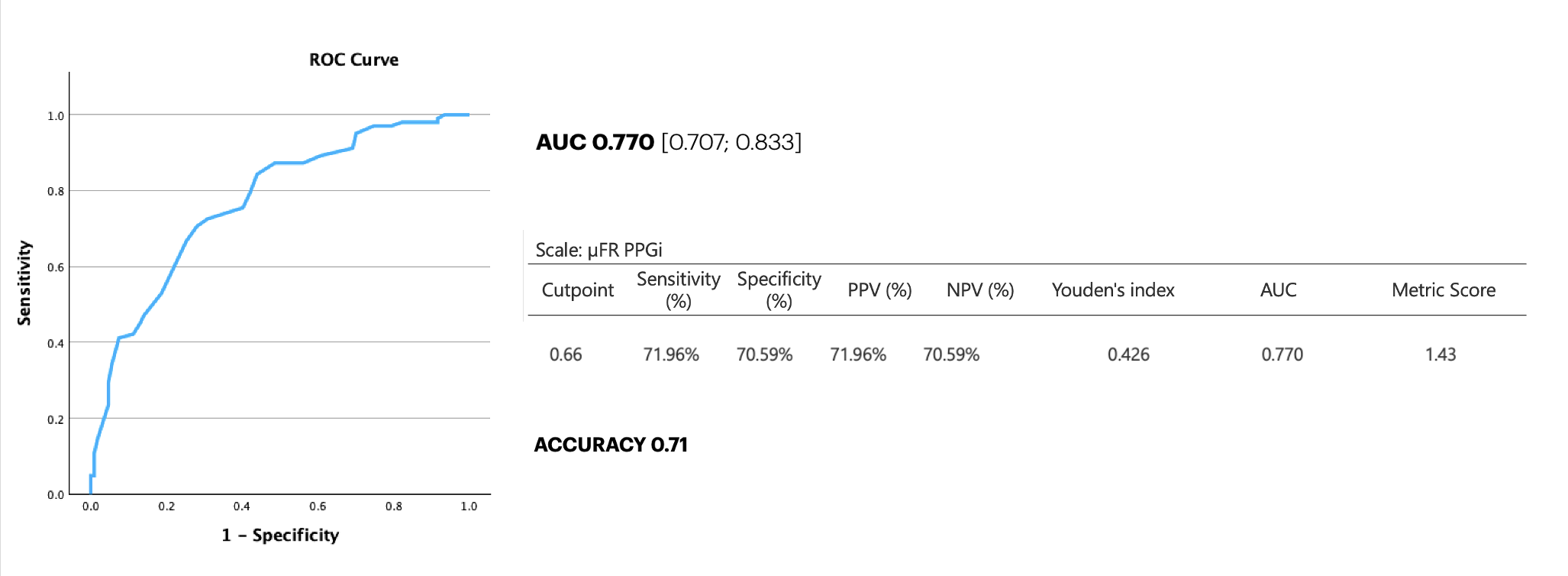
***

**Supplementary Figure 10. Agreement between iPG and µFR PPGi (Bland Altman Plot).**

iPG, iFR Pressure Gradient; µFR PPGi, µFR pullback pressure gradient index.


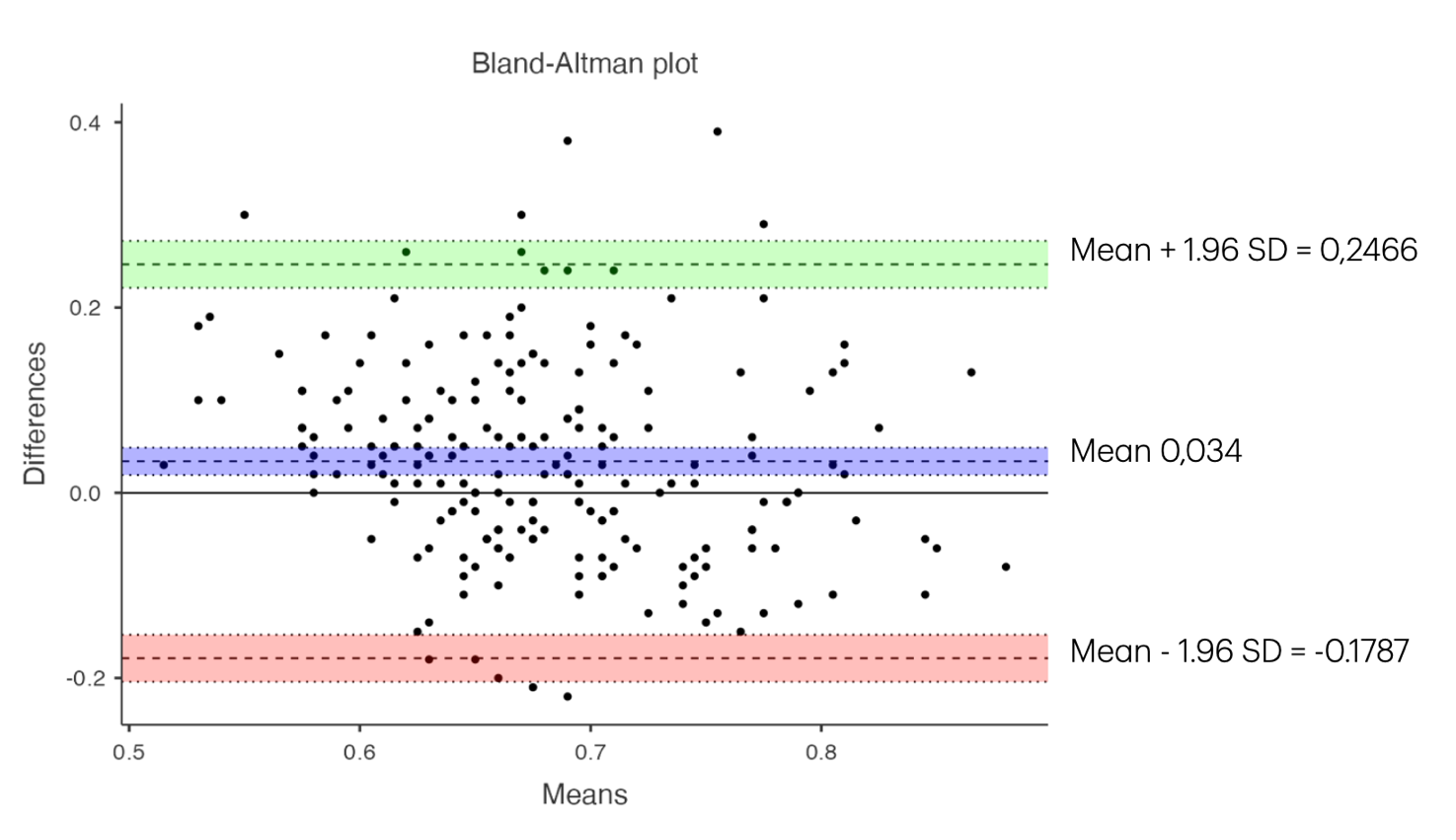

Supplement: Supplementary file 1 — Supplementary Data. [file CCD-106-2374-s001.docx]
